# The prevalence of prediabetes is high and has rapidly increased, independent of the degree of obesity, in Finnish children with overweight or obesity

**DOI:** 10.1038/s41366-025-01950-y

**Published:** 2025-11-18

**Authors:** Hanna Riekki, Linnea Aitokari, Antti Saari, Laura Kivelä, Heini Huhtala, Anna Viitasalo, Sonja Soininen, Eero A. Haapala, Timo Lakka, Kalle Kurppa

**Affiliations:** 1 Tampere Center for Child, Adolescent and Maternal Health Research, Faculty of Medicine and Health Technology, Tampere University, Tampere, Finland; 2 Celiac Disease Research Center, Faculty of Medicine and Health Technology, Tampere University, Tampere, Finland; 3 Valkeakoski Social and Health Care Centre, Wellbeing Services County of Pirkanmaa, Valkeakoski, Finland; 4 Department of Pediatrics, Kuopio University Hospital and University of Eastern Finland, Kuopio, Finland; 5 Children’s Hospital and Pediatric Research Center, University of Helsinki and Helsinki University Hospital, Helsinki, Finland; 6 Department of Pediatrics, Tampere University Hospital, Wellbeing Services County of Pirkanmaa, Tampere, Finland; 7 Faculty of Social Sciences, Tampere University, Tampere, Finland; 8 Institute of Biomedicine, University of Eastern Finland, Kuopio, Finland; 9 Physician and Nursing Services, Health and Social Services Centre, Wellbeing Services County of North Savo, Varkaus, Finland; 10 Sports and Exercise Medicine, Faculty of Sport and Health Sciences, University of Jyväskylä, Jyväskylä, Finland; 11 Department of Clinical Physiology and Nuclear Medicine, Kuopio University Hospital, Kuopio, Finland; 12 Kuopio Research, Institute of Exercise Medicine, Kuopio, Finland; 13 The University Consortium of Seinäjoki, Seinäjoki, Finland

**Keywords:** Pre-diabetes, Risk factors

## Abstract

**Objectives:**

The global increase in obesity predisposes individuals to prediabetes and type 2 diabetes, but data on their prevalence, temporal trends, and associated factors in children remain limited. We examined these issues in well-defined patient and population cohorts.

**Methods:**

Data were collected from 602 patients aged 6–16 years, who were examined in healthcare for overweight/obesity once in 2002–2020. Controls comprised 483 population-representative children aged 7–16, who participated in 1–3 prospective visits. Prediabetes signified fasting glucose 5.6–6.9 mmol/L or 2h-post-challenge glucose 7.8–11.0 mmol/L, and diabetes as values ≥7.0 mmol/L or ≥11.1 mmol/L, respectively. Factors associated with prediabetes in patients were studied using logistic regression. The prevalence of prediabetes was compared between patients having their first healthcare visit in different years between 2002 and 2019.

**Results:**

Altogether, 89.2% of patients and 3.3–4.7% of controls had obesity. The prevalence of prediabetes was 34.2% and of type 2 diabetes 1.3% among patients, and 6.9% and 0% in controls respectively, with prediabetes increasing significantly with age and stage of puberty. Both conditions were associated with presence of metabolic dysfunction-associated steatotic liver disease (OR 1.69, 95% CI 1.01–2.80) and acanthosis nigricans (1.83, 1.05–3.21), after adjusting for age. Prevalence of prediabetes increased steeply over time from 11.4% in patients examined in 2002–2004 to 50.0% in patients examined in 2017–2019 (OR 1.16, CI 1.10–1.21 *p* < 0.001) without concurrent changes in the degree of obesity, body mass index, other metabolic conditions, age, sex, or gestational/neonatal factors, except for an increase in maternal prepregnancy/pregnancy overweight (20.0–68.8%, OR 1.14, CI 1.08–1.21, *p* < 0.001).

**Conclusions:**

Prediabetes was decidedly prevalent in pediatric patients with obesity and was associated particularly with steatotic liver disease. Its prevalence increased steeply over time, independent of the degree of obesity.

## Introduction

The prevalence of obesity has increased steeply across all geographical regions [1]. Obesity is associated with metabolic disorders, including type 2 diabetes (T2D), which nowadays imposes a considerable burden on society [2]. T2D is typically preceded by moderately impaired glucose metabolism, known as prediabetes. Besides predisposing individuals to T2D, prediabetes may also be associated with adverse cardiometabolic outcomes [3]. Although previously thought to mainly affect adults, these conditions also appear to be increasingly common among children [4, 5]. The rapid progression and poor prognosis of early-onset diabetes underscore the critical need for early intervention [6].

Although some research on this issue exists, particularly data from Nordic countries and long-term trends remain limited. In the few existing reports focusing on children with overweight, the prevalence has varied widely, showing an increasing trend [7–9]. The condition also appears to be more common in males, certain ethnic groups, and individuals with metabolic dysfunction-associated steatotic liver disease (MASLD) and other metabolic risk factors [10–15]. Interpreting these results is challenging due to the use of diverse cohorts, varying prevalences of obesity, inconsistent criteria, and the lack of control groups [5, 16, 17]. The relationship between obesity and prediabetes remains partly unclear, as it may also be influenced by factors like fat tissue distribution, muscle mass, physical inactivity and maternal disorders during pregnancy [18, 19].

The Finnish population is homogeneous, and systematic healthcare data have been available for an extended period. Leveraging these advantages, we investigated the prevalence, temporal trends, and associated factors of prediabetes and T2D in children examined due to overweight and obesity. A longitudinal, population-representative cohort was available for comparisons.

## Methods

### Patients and study design

The study was conducted at Tampere University and at the University of Eastern Finland. The patients included 1000 children aged 6–16 years who received an overweight- or obesity-related ICD-10 code (E65, E66 or R63.5) in 2002–2020. They were identified from the primary and tertiary care units in the Tampere area. Medical data were collected from patient records. In total, 398 patients were excluded due to incomplete data, incorrect diagnostic codes, unavailable glucose values, or comorbidities or medications that might affect glucose metabolism. After exclusions, 602 children were included (Supplementary Fig. S1). Patient data was collected from the first obesity-related healthcare visit.

For the population cohort, 736 children aged 7–9 who started first grade in primary schools were invited to participate in the prospective Physical Activity and Nutrition in Children (PANIC) study. They did not differ in age, sex, or body mass index (BMI) Z-scores from other children in the same area [20]. Altogether, 512 attended the baseline examinations in 2007–2009, but eight were excluded due to inability to participate or lack of motivation. Comprehensive health data, along with samples, were collected at each visit [20]. The study included baseline investigations at ages 7–9, a 2-year follow-up at ages 9–11, and an 8-year follow-up at ages 14–17. Children with incomplete data and those with any diseases or treatments potentially affecting glucose metabolism, including epilepsy, hypothyroidism, type 1 diabetes, Klinefelter syndrome, ulcerative colitis, juvenile rheumatoid arthritis, multiple sclerosis, or medication for depression or neuropathic pain, were excluded. This left 483 children at baseline, 420 at the 2-year visit, and 258 at the 8-year visit. Two patients reported occasional alcohol consumption, and one control subject previous amphetamine use. This information was deemed nonsignificant.

The collection of patient data was approved by Tampere University Hospital, Tampere Healthcare Services and the Social and Health Data Permit Authority Findata, while the recruitment of children for the PANIC was approved by the Research Ethics Committee of the Hospital District of Northern Savo. Informed consent was obtained from all children prospectively enrolled, as well as from their legal guardians. The study was conducted in accordance with the Declaration of Helsinki.

### Data collection

The following data recorded during the study visit of PANIC or at the first obesity-related healthcare visit (patients) were collected: demographic and anthropometric data, stage of puberty, presence of acanthosis nigricans and chronic diseases and medications, blood pressure, use of alcohol or illicit drugs, presence of overweight, obesity and T2D in first-degree relatives, and laboratory parameters.

Growth data were converted into BMI Z-scores and weight-for-height percentages (WH%). Cut-off values for underweight, normal weight, overweight, obesity class I, and obesity class II were defined as BMI Z-scores using national cutoffs, corresponding to a BMI of <18.5, 18.5–24.9, ≥25.0, ≥30.0 and ≥35.0 kg/m^2^ at age 18 years, according to method recommended by the International Obesity Task Force (IOTF) [21, 22]. For 52 children without data of height or weight but WH% available, equivalent WH% values were used as follows: 10–20% for overweight, 20–40% for obesity class I, and >40% for obesity class II in children younger than 7 years of age; and 20–40%, 40–60%, and >60% for older children [23, 24]. These cutoffs are widely used in clinical practice in Finland [24] and, based on national data sources not publicly available, correspond to levels of body fat determined by DEXA scan, similarly to BMI Z-score thresholds. Pubertal stage was classified as prepubertal (Tanner M/G 1), pubertal (M/G 2–4), and postpubertal (M/G 5), while hypertension was defined as blood pressure exceeding the 95^th^ percentile for age, sex, and height [25].

The laboratory parameters included alanine aminotransferase (ALT), fasting glucose and insulin, total cholesterol, high-density lipoprotein (HDL) cholesterol, low-density lipoprotein (LDL) cholesterol, triglycerides, and thyroid stimulating hormone (TSH). MASLD was defined as ALT >2× the upper limit of normal in patients with overweight or obesity [13, 26]. Homeostatic Model Assessment for Insulin Resistance (HOMA-IR) was calculated as fasting insulin mIU/mL × glucose mmol/L/22.5 [27]

Perinatal data on the patients were retrieved to investigate potential temporal changes in these measures that might help explain the possible increase in prediabetes prevalence. Maternal data included presence of gestational hypertension, diabetes, pre-eclampsia, and abnormal weight gain as diagnosed by clinicians, as well as smoking and prepregnancy or gestational overweight or obesity [28]. Offspring data included gestational age and presence of macrosomia (birth weight >4.5 kg), large for gestational age (LGA), and small for gestational age (SGA). Data for analyzing temporal changes in gestational and neonatal risk factors between 2002 and 2019 were available for 344–380 patients, except for gestational diabetes and weight gain, for which data were available for 119 and 191 patients, respectively.

### Prediabetes and type 2 diabetes

Prediabetes was defined as fasting glucose levels 5.6–6.9 mmol/L, also called impaired fasting glucose (IFG), or 2-h levels 7.8–11.0 mmol/L in the oral glucose tolerance test (OGTT), also known as impaired glucose tolerance (IGT). T2D was defined as fasting glucose ≥7.0 mmol/L or 2-h glucose >11.0 mmol/L as recommended by the American Diabetes Association [3]. Glycated hemoglobin (HbA1c) was unavailable for the controls and was therefore not included in the analyses.

### Statistical methods

Categorical variables are reported as numbers and percentages. The distributions of some continuous variables were skewed, and they are therefore reported as medians and quartiles. The prevalences of prediabetes and T2D were calculated separately for ages <9, 9–11, and ≥12. The characteristics of patients with or without prediabetes and T2D were compared with the Mann–Whitney test for continuous variables and with the Chi-square test for dichotomous or categorical variables. Spearman’s coefficients for correlation were used to analyze the association between BMI Z-score and fasting glucose in the controls. The associations of prediabetes and T2D with patient characteristics were analyzed using logistic regression, both unadjusted and adjusted for age. ALT was included as a continuous variable in the logistic regression analysis; however, to enhance clarity, some of the results are presented per ten units, as one unit is too small to effectively display the findings. The exact characteristics of patients with T2D are not presented separately due to the low number of these cases and strict national data privacy regulations. The associations between the year of the first obesity-related visit (as a continuous variable) and the prevalence of prediabetes or T2D, female sex, obesity class I or II, hypertension, dyslipidemia, and MASLD were examined using logistic regression, while those between year of examination and median age, BMI-Z score, ALT, triglycerides, total-, LDL- and HDL cholesterol, fasting glucose and median insulin were examined by Pearson correlation among the patients. The association between the prevalence of prediabetes/T2D and year of examination was further analyzed using logistic regression models adjusted for median age, sex, BMI Z-score, ALT, hypertension, or dyslipidemia as a univariate model and also adjusted for age, sex and BMI z-score as a multivariable model. The analysis was not adjusted for perinatal factors due to the limited number of participants with available data. The association between the prevalence of prediabetes or T2D and year of examination was also analyzed separately in patients from primary or specialized healthcare. Temporal trends in prenatal variables were analyzed using Pearson correlation and logistic regression. The year 2020 was excluded from the analyzes due to the low number of participants. Statistical significance was defined as *P*-value < 0.05. Analyses were performed using SPSS, version 25.0 (IBM Corp., Armonk, NY, USA).

## Results

### Basic characteristics and prevalence of prediabetes and type 2 diabetes

The median age of the patients was 11.6, with 41.0% being girls (Table 1). The median ages of the controls were 7.6 at baseline, 9.8 at 2-year follow-up, and 15.8 at 8-year follow-up. Of the controls in these three study stages, 44.6–48.8% were girls, 3.3–4.7% had obesity, and 13.9–16.7% had overweight.

**Table 1:** Characteristics of the patient cohort of 602 children with overweight or obesity, as a whole group and divided by the presence or absence of prediabetes or type 2 diabetes (T2D).

Continuous variables	Data	All children		Prediabetes/ T2D^a^				P value^b^
		*n* = 602		Yes, *n* = 206		No, *n* = 396		
		Median	Quartiles	Median	Quartiles	Median	Quartiles	
Age, years	602	11.6	9.5, 13.8	12.6	11.1, 4.5	11.0	8.7, 13.1	**<0.001**
Body mass index Z-score	550	2.4	2.2, 2.7	2.4	2.1, 2.7	2.4	2.2, 2.7	0.918
HOMA-IR	241	5.9	2.9, 7.3	8.2	5.2, 8.9	4.6	2.4, 5.7	**<0.001**
Insulin, mU/L	243	24	12.0, 29.0	31.4	19.0, 35.5	19.8	11.0, 24.0	**<0.001**
Total cholesterol, mmol/L	546	4.4	3.8, 4.9	4.3	3.8, 4.8	4.4	3.9, 4.9	0.189
Triglycerides, mmol/L	539	1.2	0.7,1.5	1.3	0.8, 1.5	1.2	3.9, 4.9	**0.006**
LDL cholesterol, mmol/L	536	2.7	2.2, 3.2	2.7	2.2, 3.2	2.7	2.3, 3.2	0.870
HDL cholesterol, mmol/L	541	1.2	1.0, 1.4	1.2	1.0, 1.4	1.3	1.0, 1.4	**0.018**
ALT, U/L	544	33.0	18.0, 35.0	38.0	19.0, 39.0	30.4	18.0, 33.0	**0.015**
TSH, mU/L	466	3.0	1.9, 3.7	3.0	2.0, 3.7	3.0	1.9, 3.7	0.946
Categorical variables	Data	*n*	%	*n*	%	*n*	%	P value^b^
Girls	602	247	41.0	79	38.3	168	42.4	0.335
Obesity class I or higher^c^	602	537	89.2	183	88.8	354	89.4	0.834
Obesity class II or higher^c^	602	259	43.0	89	43.2	170	42.9	0.949
Acanthosis nigricans	600	59	9.8	31	15.1	28	7.1	**0.002**
Hypertension^d^	494	233	47.2	83	47.4	150	47.0	0.931
MASLD^e^	544	75	13.8	37	19.5	38	10.7	**0.005**
Overweight/obesity in FDR	230	198	86.1	67	83.8	131	87.3	0.455
Type 2 diabetes in FDR	314	57	18.2	26	22.8	31	15.5	0.106

*MASLD* metabolic dysfunction-associated steatotic liver disease, *ALT* alanine aminotransferase, *FDR* first-degree relative, *HDL* high-density lipoprotein, *HOMA-IR* Homeostatic Model Assessment of Insulin Resistance, *LDL* low-density lipoprotein, *OGTT* oral glucose tolerance test, *TSH* thyroid stimulating hormone.

Laboratory measurements excluding TSH and ALT are fasting samples. Values in bold face denote statistical significance.

^a^Defined based on increased glucose value either on fasting sample or in 2 h oral glucose tolerance test utilizing cutoffs recommended by the American Diabetes Association [3].

^b^Between prediabetes/type 2 diabetes and normoglycemia.

^c^As defined by Cole et al. [22] and Saari et al. [21].

^d^Blood pressure >95^th^ percentile as defined by Flynn et al. [25].

^e^As defined by Eslam et al. [26].

**Fig. 1:**
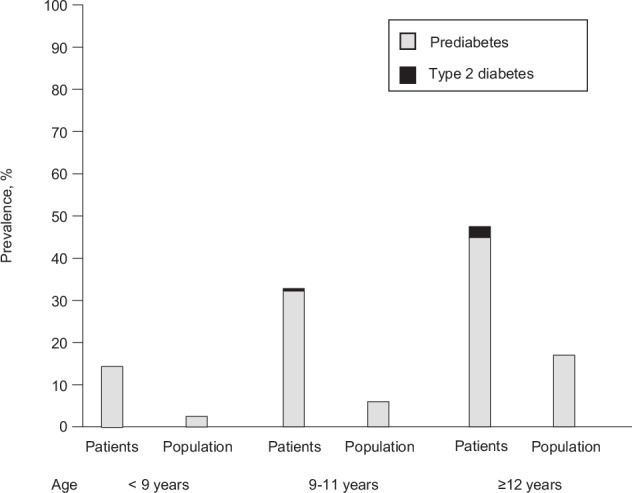
Prevalence of prediabetes and type 2 diabetes in a patient cohort of 602 children with overweight/obesity and population cohort, divided into three different age groups (*n* = 483, *n* = 420 and *n* = 258). Prediabetes was defined based fasting glucose or 2 h oral glucose tolerance test, utilizing cutoffs by the American Diabetes Association [3].

Of the patients, 34.2% had prediabetes and 1.3% T2D. IGT was diagnosed in 27.0% (89 tested with OGTT), and IFG was found in 32.7% based on data from 602 patients. The combined prevalence of prediabetes and T2D was similar in patients from primary healthcare and specialized care (32.7% vs. 35.0%, *p* = 0.572). Altogether, 6.9% of the controls had prediabetes, and none had T2D. The prevalence of prediabetes and T2D increased with age in both patients and controls (Fig. 1), as well as with pubertal stage in the patients (22.8% in prepuberty, 43.9% in puberty, 49.3% in postpuberty; *p* < 0.001) and controls (3.7%, 13.5% and 14.3%, *p* < 0.001). In the controls, fasting glucose correlated with BMI Z-score (*r* = 0.114, *p* < 0.001). The prevalence of prediabetes was 6.4% in controls with normal weight, 6.9% in those with overweight, and 18.2% in those with obesity (*p* = 0.010).

### Temporal trends of glucose disturbances and early risk factors in patients

**Fig. 2:**
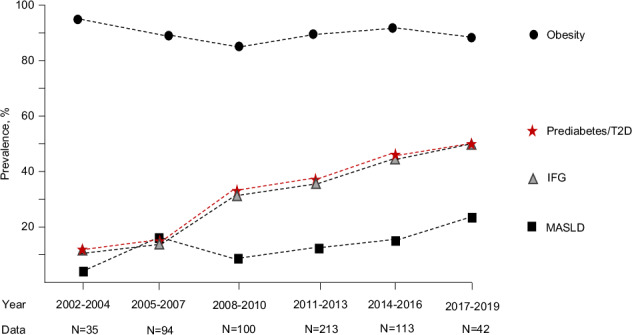
Temporal trends in the prevalence of obesity, prediabetes/type 2 diabetes, impaired fasting glucose (IFG) and metabolic dysfunction-associated steatotic liver disease (MASLD) in 597 children with overweight/obesity who had their first obesity-related healthcare visit in different years. Changes in sex distribution (OR for female sex 1.02, 95% CI 0.98–1.07, *p* = 0.349), median ages (*r* = −0.037, *p* = 0.373) and median BMI z-scores in different years of examination (2.47 [2002–4], 2.54 [2005–7], 2.36 [2008–10], 2.39 [2011–13], 2.46 [2014–16], 2.47 [2017–19], *r* = 0.005, *p* = 0.901) were not significant. Prediabetes was defined based fasting glucose or 2 h oral glucose tolerance test, utilizing cutoffs by the American Diabetes Association [3].

The prevalences of prediabetes or T2D and IFG were higher in patients making their first obesity-related healthcare visit in 2017–2019 compared to earlier years, while no significant changes were observed in the prevalence of obesity or MASLD (Fig. 2).

There was also a significant positive association between the year of the first obesity-related visit and the prevalence of prediabetes or T2D (OR 1.16, 95% CI 1.10–1.21, *p* < 0.001), level of fasting glucose (*r* = 0.191, *p* < 0.001) and median insulin (*r* = 0.211, *p* < 0.001). The increasing prevalence of prediabetes/T2D over time were observed in both primary (OR 1.18, 95% CI 1.06–1.33, *p* = 0.004) and specialized care (1.19, 1.12–1.26, *p* < 0.001), and remained significant after adjustment for age, sex, BMI z-score, ALT, hypertension and dyslipidemia (Supplementary Table S1). Significant association between the year of the examination and prediabetes/T2D prevalence persisted in a multivariable model consisting of examination year, children sex, age and BMI Z-score (OR 1.18, 95% CI 1.12–1.25, *p* < 0.001, Supplementary Table S1).

A significant negative association was observed between the year of the first obesity-related visit and the level of total cholesterol (*r* = –0.168, *p* < 0.001), and in the prevalence of hypertension (OR 0.940, 95% CI 0.894–0.988, *p* = 0.015). No changes over time were seen in child’s age at the obesity-related healthcare visit, BMI z-score, ALT, triglycerides, LDL, HDL, or in prevalence of female sex, obesity class I or II, dyslipidemia, or MASLD (Supplementary Table S2).

The prevalence of maternal overweight or obesity increased from 20.0% to 68.8% in patients making their obesity-related healthcare visit between 2002 and 2019 (OR 1.14, 95% CI 1.08–1.21, *p* < 0.001), while there were no changes in SGA, LGA, smoking, or hypertension (Supplementary Fig. S2, Supplementary Table S3). No changes were observed in the prevalence of gestational diabetes, pre-eclampsia, gestational weight gain, macrosomia, gestational age, or birth size (Supplementary Table S3). The prevalence of maternal overweight/obesity did not differ significantly between children with and without prediabetes (45.0% vs 38.8%, *p* = 0.240).

### Associated factors for prediabetes/type 2 diabetes in the patient cohort

Patients with prediabetes or T2D were older, had higher levels of fasting insulin, HOMA-IR, triglycerides, and ALT, as well as lower HDL cholesterol level. They were also more likely to have acanthosis nigricans and MASLD compared to those without prediabetes or T2D (Table 1). Unadjusted and age-adjusted associations between prediabetes or T2D and other variables are presented in Table 2.

**Table 2:** Factors associated with prediabetes and type 2 diabetes^a^ in the patient cohort of 602 children with overweight or obesity, analyzed using univariate logistic regression and models adjusted for age.

Continuous variables	Unadjusted	Adjusted for age
	OR (95% CI)	OR (95% CI)
Age, years	**1.26 (1.17–1.35)**	N/A
Body mass index Z-score	1.03 (0.70–1.53)	
Total cholesterol, mmol/L	0.86 (0.70–1.05)	
Triglycerides, mmol/L	**1.34 (1.04–1.73)**	1.12 (0.86–1.45)
LDL cholesterol, mmol/L	0.99 (0.80–1.23)	
HDL cholesterol, mmol/L	0.56 (0.31–1.02)	
ALT, 10 U/L	**1.09 (1.02–1.15)**	1.06 (1.00–1.13)
TSH, mU/L	0.95 (0.90–1.12)	
**Categorical variables**		
Female sex	1.84 (0.60–1.19)	
Hypertension^b^	1.02 (0.70–1.47)	
Type 2 diabetes in FDR	1.61 (0.90–2.88)	
Overweight or obesity in FDR	0.75 (0.35–1.61)	
Acanthosis nigricans	**2.34 (1.36–4.02)**	**1.83 (1.05–3.21)**
Pubertal stage:		
Pubertal^c^	**2.66 (1.78–4.0)**	N/A
Postpubertal^d^	**3.29 (1.91–5.68)**	N/A
MASLD^e^	**2.01 (1.23–3.29)**	**1.69 (1.01–2.80)**

Factors were analyzed using either female sex, no hypertension, no type 2 diabetes, no overweight/obesity, no acanthosis nigricans, no MASLD, ALT ≤80 U/L or prepubertal stage as the reference (=odds ratio 1.0).

Values in bold face denote statistical significance. All lipids are fasting values. The association between prediabetes or type 2 diabetes and pubertal stage were not adjusted for age due to collinearity.

*ALT* alanine aminotransferase, *CI* confidence interval, *FDR* first-degree relative, *HDL* high-density lipoprotein, *LDL* low-density lipoprotein, *MASLD* metabolic dysfunction-associated steatotic liver disease, *N/A* not applicable, *OR* odds ratio, *TSH* thyroid stimulating hormone.

^a^Defined based on increased glucose value either on fasting sample or in 2 h oral glucose tolerance test utilizing cutoffs recommended by the American Diabetes Association [3].

^b^Blood pressure >95^th^percentile as defined by Flynn et al. [25].

^c^Tanner M/G 2–4.

^d^Tanner M/G 5.

^e^As defined by Eslam et al. [26].

Patients with T2D had more advanced puberty, lower HDL cholesterol, a higher prevalence of acanthosis nigricans and MASLD, and a higher prevalence of T2D in first degree relatives compared to those without it (data not shown due to strict national data privacy regulations).

## Discussion

We observed the prevalence of prediabetes of 34.2% and of T2D of 1.2% in patients with overweight or obesity, compared to 6.9% and 0%, respectively in controls. For comparison, a meta-analysis reported a global prediabetes prevalence of 8.8% in children, with the highest rates in Africa and the Americas and the lowest in Europe. The figure increased to 9.8% in children with overweight and to 14.3% in those with obesity [29]. Overall, there is substantial variation between studies. For example, Andes et al. observed a prevalence of 18.8% in the NHANES study, which increased to 25.7% in those with obesity [30]. Ghaddar et al. reported respective figures of 26.2% and 31% in 7–12-year-old US children [8]. In Europe, Brufani et al. detected glucose dysregulation in 12.4% of 3–18-year-old Italian children with overweight or obesity, while Hagman et al. reported this in 5.7% of 2–18-year-old German children and 17.1% of Swedish children with obesity [7, 31].

Obesity is a primary risk factor for glucose dysregulation; therefore, its prevalence and severity are likely to account for much of the variation in the prevalence of prediabetes. Obesity was substantially less common in our controls (3.3–4.7%) than in the US population (16.2–19.2%), likely partially explaining the similarly wide differences in prediabetes prevalence between the countries [8, 30]. Although many studies show an almost linear increase in the likelihood of glucose abnormalities with degree of obesity [8, 31, 32], this trend was not evident here. A potential explanation is the high proportion of children with obesity class II in this cohort, which may also account for the high prevalence of prediabetes compared to controls, who – by global comparison – tend to have relatively low rates of overweight and obesity.

Inconsistent definitions of prediabetes could also impact the comparisons. Here, it was defined based on IFG or IGT, whereas other studies may have used different criteria, such as combinations of IFG, IGT, and HbA1c [7, 8, 30, 31]. These measures reflect different aspects of glucose dysregulation and have varying prognostic significance and sources of error [30, 32]. The lack of HbA1c could be considered a weakness, although its utility in children is debated [33, 34]. Another controversial issue is the choice of the cut-off values, which have sometimes been criticized for being too low [35]. However, their usefulness as markers of disturbed glucose metabolism was demonstrated by the differences between patients and controls, as well as between controls with normal and increased weight. Moreover, early detection of prediabetes may enable timely interventions, reducing progression to T2D. Additional studies, for instance those using continuous glucose monitoring, could establish optimal cutoffs for identifying children at increased risk - without leading to unnecessary burden on families.

Besides obesity, other characteristics associated with prediabetes may also vary between cohorts. In line with earlier reports, we observed that the risk of prediabetes increased with age [7, 8, 30, 31]. Furthermore, both we and Brufani et al. identified an association with advanced pubertal stage, which aligns with the phenomenon of transient insulin resistance [7, 36]. Intriguingly, this may not fully resolve in adolescents with obesity, possibly explaining why prediabetes continued to increase from puberty to postpuberty [37]. Additionally, sex differences in the timing and magnitude of pubertal insulin resistance may contribute to the reported overrepresentation of prediabetes in boys [30, 31]. However, we, along with some others, found no sex difference in the prevalence, indicating the need for further research [7, 8].

Ethnicity partly explains the variation in the prevalence of prediabetes, with the risk gradually decreasing from non-Hispanic Blacks to non-Hispanic White [16, 38]. Consequently, the aforementioned high-prevalence US cohort predominantly consisted of Hispanic and non-Hispanic Blacks, among whom prediabetes was 2.8–4.8 times more common than among non-Hispanic Whites [8]. Conversely, Andes et al. included mostly non-Hispanic Whites, among whom the prevalence was lower as a whole even among those with obesity than in the study by Ghaddar et al. [8, 30]. Ethnicity data were lacking in the European studies, but they likely included mostly non-Hispanic White [7, 31]. However, the prevalence was markedly higher in Sweden than in Germany, and even higher in our cohort, which likely comprised mostly non-Hispanic White, despite comparable BMI [31]. This could be due to genetic variation and unidentified environmental factors. Altogether, glucose disturbances appear to result from a complex interaction between genetics, the quantity and function of fat and muscular tissue, physical activity, diet, and other factors [18, 19].

Additional factors associated with prediabetes included higher levels of triglycerides and ALT, and presence of acanthosis nigricans and MASLD, most of which were independent of age. This is in line with the reported clustering of glucose disturbances and other cardiometabolic risk factors already in adolescence [10, 11, 15]. Of particular interest is the association between MASLD, which, as also noted here, even occurs independent of the degree of obesity [14, 32, 39]. This, together with the key role of liver in glucose metabolism, indicates shared pathogenesis [40]. In line, the study by Putri et al. found that presence of MASLD markedly increased the risk of T2D, and a synergistic effect on risk was observed in children with obesity, MASLD, and prediabetes [41]. The risk of such a vicious circle further supports screening for MASLD in children with prediabetes, and vice versa.

We observed a striking increase in prediabetes over time, independent of changes in BMI z-score or other metabolic conditions. In fact, the prevalence of hypertension and levels of total cholesterol even decreased during the same period, while age, sex distribution, prevalence of obesity class I or II, and other metabolic disturbances remained unchanged. We cannot fully exclude lower-threshold testing or changes in referral practice, which may partly explain the observed increase in specialized care. However, we consider these factors unlikely to fully account for the observed changes of this magnitude. To support this conclusion, a similar increase was also observed in primary healthcare, which patients can access without a referral. Additionally, to the best of our knowledge, there was also no changes in the laboratory methods and reference ranges used. Nevertheless, it must be emphasized that these results were observed in a single population, and further studies are needed to better understand the trend.

Generally, an increase in prediabetes has been observed also previously, the main explanation being the obesity epidemic [9, 29]. Mayer-Davis et al. reported gradual increase in pediatric T2D in 2002–12, but noted that there was no similar change in obesity within the population [42]. Our novel finding of an obesity-independent change could be due to population-level changes, for example, in body composition, diet and physical activity. An additional contributing factor could be early-life programming, as maternal obesity tripled during the same period [43]. However, no differences were observed between children with and without prediabetes regarding birth size or prevalence of gestational diabetes, although data on the latter were limited. There was also a parallel, albeit non-significant, increase in MASLD, which is an important consideration due to its strong association with T2D. Of note, although these findings are likely generalizable to the homogenous Finnish population, further studies in more diverse populations and geographical areas are needed. Additionally, due to the short recruitment period for the baseline visit, it is not possible to determine whether a similar increase in prediabetes occurred also in the PANIC cohort. Overall, our findings add to concerns about the already alarming predictions regarding the future prevalence of T2D [44].

The study consisted of a large cohort of patients from different levels of healthcare and a population-representative control cohort. However, the somewhat higher proportion of children from tertiary care than primary care, along with the high prevalence of class II obesity, may have led to an overestimation of the prediabetes prevalence. In contrast, the lower prevalence of obesity in the PANIC cohort compared to the general Finnish population may have resulted in an underestimation of glucose disturbances [20, 45, 46]. A further strength was the careful consideration of confounding factors. Systematic recording of glucose measurements throughout the study period also enabled the assessment of temporal trends. Limitations include the retrospective design in the patient cohort, which led to incomplete OGTT measurements, and lack of HBA1c values for the controls. Furthermore, the statistical power was reduced in some subgroup analyses, including the prevalence of MASLD across different time intervals. Finally, while the availability of free public healthcare and the homogeneous study population increased internal validity, these factors may limit the generalizability of the results.

## Conclusion

Disturbances in glucose metabolism were highly prevalent among pediatric patients with overweight or obesity and were particularly associated with MASLD. Additionally, the prevalence of prediabetes appears to be increasing rapidly, independent of changes in the degree of obesity or other metabolic conditions. This alarming trend warrants further confirmation, along with an in-depth investigation into its underlying causes.

## Supplementary information

Supplementary Table 1

Supplementary Table 2

Supplementary Table 3

Supplementary Figure legends

Supplementary Figure 1

Supplementary Figure 2

## Data Availability

Due to privacy and confidentiality concerns, the data supporting the findings of this study are not publicly available. De-identified data are available from the corresponding author upon reasonable request.
