# Supplementary material for: The prevalence of prediabetes is high and has rapidly increased, independent of the degree of obesity, in Finnish children with overweight or obesity
Source: Int J Obes (Lond). 2025 Nov 18;50(2):407–13. doi: 10.1038/s41366-025-01950-y (PMC12913023; doi:10.1038/s41366-025-01950-y)
Supplement: Supplementary file 1 — Supplementary Table 1 [file 41366_2025_1950_MOESM1_ESM.docx]

| **Table S1**. The association between the year of the first obesity-related visit and the prevalence of prediabetes or type 2 diabetes^1^ in a patient cohort of 597 children with overweight/obesity visiting healthcare between 2002-2019. | | | | |
| --- | --- | --- | --- | --- |
|  | Data available | OR | 95% CI | P value |
| Unadjusted odds for examination year of prediabetes/T2D | 597 | 1.16 | **1.10**–**1.21** | **<0.001** |
| *Univariate model adjusted for:* |  |  |  |  |
| Age, years | 597 | 1.18 | **1.12**–**1.25** | **<0.001** |
| Sex | 597 | 1.16 | **1.10**–**1.22** | **<0.001** |
| Body mass index Z-score | 545 | 1.16 | **1.10**–**1.22** | **<0.001** |
| ALT, U/l | 541 | 1.17 | **1.11**–**1.24** | **<0.001** |
| Hypertension^2^ | 490 | 1.17 | **1.10**–**1.24** | **<0.001** |
| Borderline dyslipidemia^3^ | 543 | 1.17 | **1.10**–**1.23** | **<0.001** |
| Significant dyslipidemia^3^ | 543 | 1.17 | **1.11**–**1.23** | **<0.001** |
| *Odds for examination year in multivariable model including* *year of the examination, age, sex and BMI Z-score* | 545 | 1.18 | **1.12-1.25** | **<0.001** |
| The association between the examination year and prediabetes/T2D prevalence was calculated with univariate logistic regression using time as continuous variable, model adjusted either with age, sex, BMI Z-score, ALT, hypertension or dyslipidemia, as well as multivariable model.  ^1^Defined based on increased glucose value either on fasting sample or in 2h oral glucose tolerance test utilizing cutoffs recommended by the American Diabetes Association (3); ^2^Blood pressure >95^th^percentile as defined by Flynn et al. (25); ^3^Any lipid abnormality with cutoffs as defined by de Jesus et al. (46). ALT, alanine aminotransferase; CI, confidence interval; OR, odds ratio. Values in bold face denote statistical significance. Associations were analyzed using univariate logistic regression and models adjusted for age, sex, BMI z-score and ALT, or for the presence of hypertension, or mild or moderate dyslipidemia. | | | | |
