# Supplementary material for: The prevalence of prediabetes is high and has rapidly increased, independent of the degree of obesity, in Finnish children with overweight or obesity
Source: Int J Obes (Lond). 2025 Nov 18;50(2):407–13. doi: 10.1038/s41366-025-01950-y (PMC12913023; doi:10.1038/s41366-025-01950-y)
Supplement: Supplementary file 2 — Supplementary Table 2 [file 41366_2025_1950_MOESM2_ESM.docx]

| **Table S2**. The association between the year of the first obesity-related visit and the patient characteristics or other metabolic comorbidities in a patient cohort of 597 children with overweight/obesity visiting healthcare between 2002-2019. | | | | |
| --- | --- | --- | --- | --- |
| **Continuous variables** | Data available | r | | P value |
| Age | 597 | -0.037 | | 0.373 |
| Body mass index Z-score | 545 | 0.005 | | 0.901 |
| ALT | 541 | 0.034 | | 0.431 |
| Triglycerides | 535 | -0.064 | | 0.142 |
| Total cholesterol | 542 | **-0.168** | | **<0.001** |
| LDL cholesterol | 532 | -0.005 | | 0.902 |
| HDL cholesterol | 537 | 0.004 | | 0.931 |
|  |  |  | |  |
| **Dependent categorial variables** | Data available | OR | CI | P value |
| Female sex | 597 | 1.02 | 0.98–1.07 | 0.349 |
| Obesity class I^1^ | 597 | 1.00 | 0.93–1.07 | 0.944 |
| Obesity class II^1^ | 597 | 1.01 | 0.97–1.05 | 0.658 |
| Hypertension^2^ | 490 | 0.94 | **0.89**–**0.99** | **0.015** |
| Borderline dyslipidemia^3^ | 543 | 0.97 | 0.91–1.02 | 0.237 |
| Significant dyslipidemia^3^ | 543 | 0.97 | 0.93–1.02 | 0.202 |
| MASLD^4^ | 541 | 1.04 | 0.97–1.12 | 0.253 |
| For continuous variables, the association was examined by Pearson correlation, and for categorical variables by logistic regression. ^1^As defined by Cole et al. (22) and Saari et al. (21); ^2^Blood pressure >95^th^percentile as defined by Flynn et al. (25); ^3^Any lipid abnormality with cutoffs defined by de Jesus et al. (46); ^4^As defined by Eslam et al. (26). ALT, alanine aminotransferase; CI, confidence interval; HDL, high-density lipoprotein; LDL, low-density lipoprotein; MASLD, metabolic dysfunction‐associated steatotic liver disease; OR, odds ratio; r, correlation coefficient. Values in bold face denote statistical significance. | | | | |
