# Supplementary material for: The prevalence of prediabetes is high and has rapidly increased, independent of the degree of obesity, in Finnish children with overweight or obesity
Source: Int J Obes (Lond). 2025 Nov 18;50(2):407–13. doi: 10.1038/s41366-025-01950-y (PMC12913023; doi:10.1038/s41366-025-01950-y)
Supplement: Supplementary file 3 — Supplementary Table 3 [file 41366_2025_1950_MOESM3_ESM.docx]

| **Table S3**. The association between the year of the first obesity-related visit and perinatal factors in a patient cohort of 597 children with overweight/obesity visiting healthcare between 2002-2019. | | | | |
| --- | --- | --- | --- | --- |
| **Continuous variables** | Data available | r | | P value |
| Gestational age, weeks | 380 | -0.054 | | 0.282 |
| Gestational weight gain, kg | 191 | 0.075 | | 0.303 |
| Birth weight, SD | 377 | 0.088 | | 0.077 |
| Birth height, SD | 377 | 0.089 | | 0.072 |
| Head circumference, SD | 344 | 0.082 | | 0.142 |
|  |  |  | |  |
| **Dependent categorical variables** | Data available | OR | CI | P value |
| Maternal overweight or obesity^1^ | 380 | **1.14** | **1.08**–**1.21** | **<0.001** |
| Smoking during gestation | 380 | 1.00 | 0.95–1.06 | 0.988 |
| Gestational hypertension^1^ | 380 | 1.11 | 0.95–1.29 | 0.181 |
| Gestational diabetes^1^ | 119 | 1.03 | 0.93–1.13 | 0.596 |
| Pre-eclampsia^1^ | 380 | 1.03 | 0.92–1.15 | 0.610 |
| Birth weight >4500g | 377 | 1.05 | 0.93–1.19 | 0.411 |
| LGA | 377 | 1.05 | 0.95–1.16 | 0.316 |
| SGA | 377 | 0.86 | 0.73–1.00 | 0.055 |
| For continuous variables, the association was examined by Pearson correlation, and for categorical variables by logistic regression. ^1^Diagnosed by the clinician in charge. CI, confidence interval; LGA, large for gestational age, birth weight >2 SD; OR, odds ratio for time; r, correlation coefficient; SD, standard deviation based on national reference data; SGA, small for gestational age, birth weight <2 SD. Values in bold face denote statistical significance. | | | | |
