# Supplementary material for: The prevalence of prediabetes is high and has rapidly increased, independent of the degree of obesity, in Finnish children with overweight or obesity
Source: Int J Obes (Lond). 2025 Nov 18;50(2):407–13. doi: 10.1038/s41366-025-01950-y (PMC12913023; doi:10.1038/s41366-025-01950-y)
Supplement: Supplementary file 4 — Supplementary Figure legends [file 41366_2025_1950_MOESM4_ESM.docx]

**Supplementary Figure S1.** Flowchart of the patient cohort consisting of 602 children and adolescents with overweight or obesity.

**Supplementary Figure S2.** Temporal trends in the prevalence of prepregnancy/pregnancy overweight or obesity, gestational smoking, hypertension, large for gestational age (LGA) and small for gestational age (SGA) in 380 children-mother pairs from the patient cohort of children and adolescents with overweight or obesity examined in different years.
