# Supplementary material for: The prevalence of prediabetes is high and has rapidly increased, independent of the degree of obesity, in Finnish children with overweight or obesity
Source: Int J Obes (Lond). 2025 Nov 18;50(2):407–13. doi: 10.1038/s41366-025-01950-y (PMC12913023; doi:10.1038/s41366-025-01950-y)
Supplement: Supplementary file 5 — Supplementary Figure 1 [file 41366_2025_1950_MOESM5_ESM.pptx]

## Slide 1
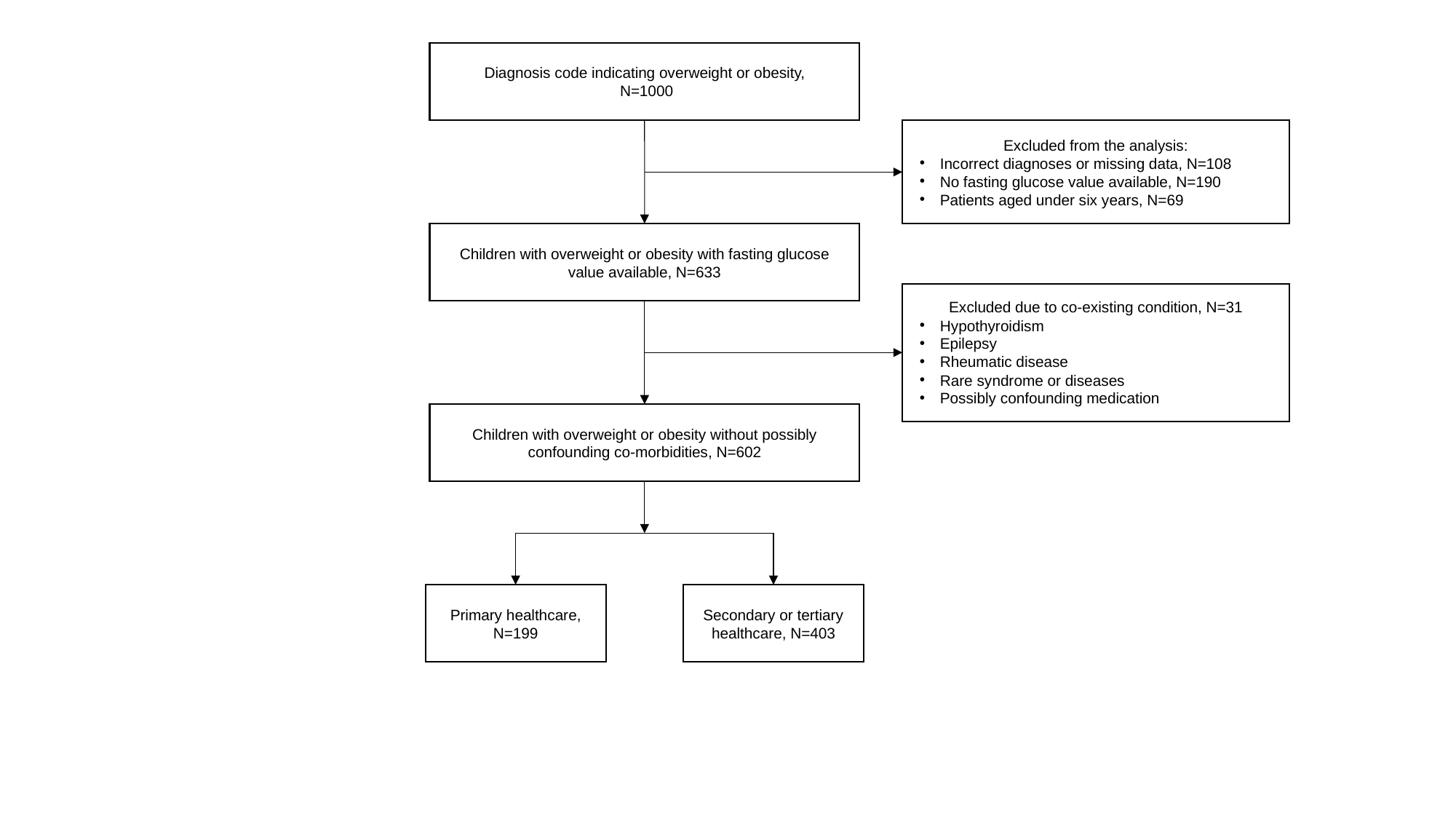

Diagnosis code indicating overweight or obesity,
 N=1000
Excluded from the analysis:
Incorrect diagnoses or missing data, N=108
No fasting glucose value available, N=190
Patients aged under six years, N=69
Children with overweight or obesity with fasting glucose value available, N=633
Excluded due to co-existing condition, N=31
Hypothyroidism
Epilepsy
Rheumatic disease
Rare syndrome or diseases
Possibly confounding medication
Children with overweight or obesity without possibly confounding co-morbidities, N=602
Primary healthcare, N=199
Secondary or tertiary healthcare, N=403
