# Supplementary material for: The prevalence of prediabetes is high and has rapidly increased, independent of the degree of obesity, in Finnish children with overweight or obesity
Source: Int J Obes (Lond). 2025 Nov 18;50(2):407–13. doi: 10.1038/s41366-025-01950-y (PMC12913023; doi:10.1038/s41366-025-01950-y)
Supplement: Supplementary file 6 — Supplementary Figure 2 [file 41366_2025_1950_MOESM6_ESM.pptx]

## Slide 1
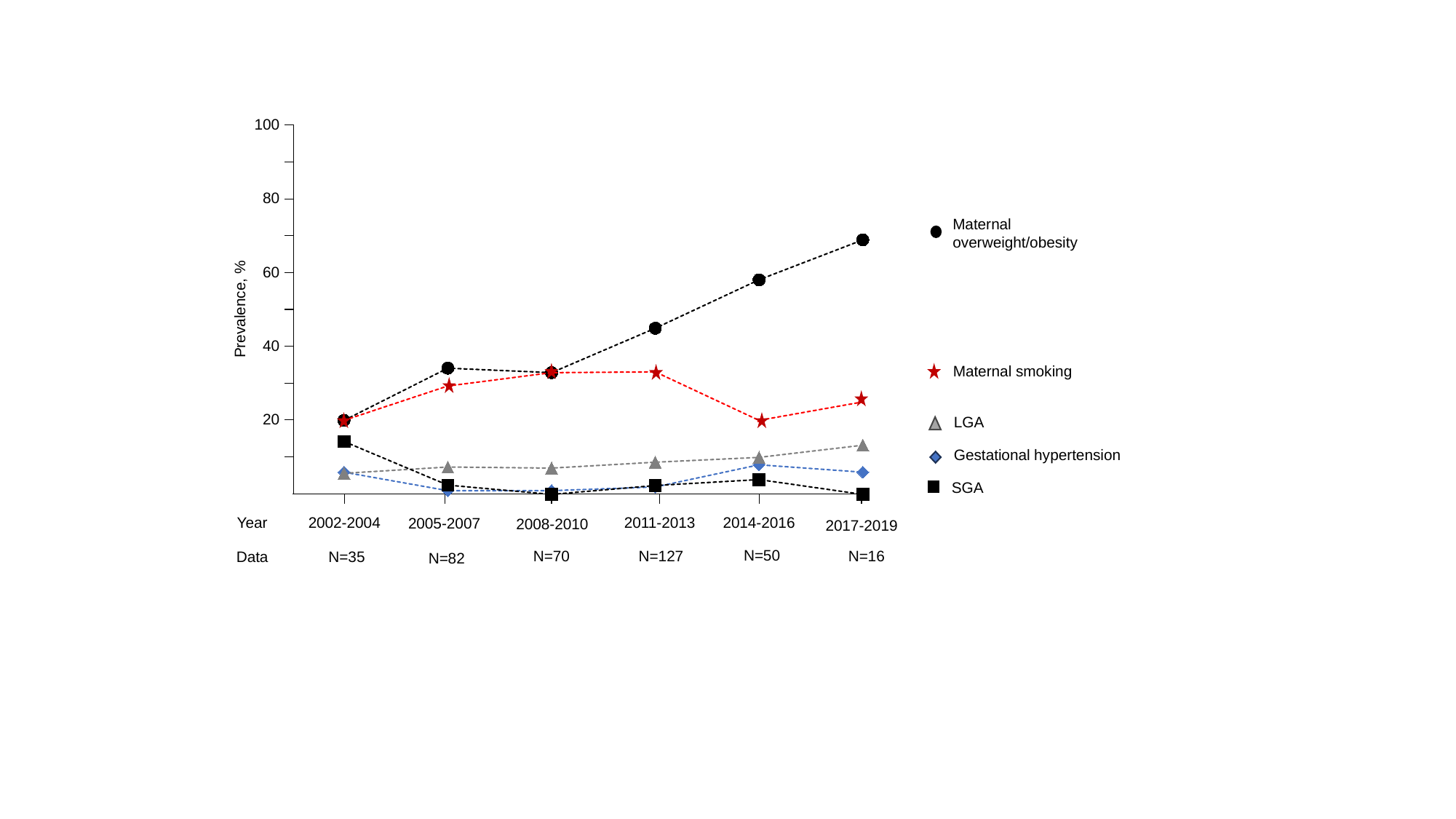

100
80
60
40
20
### Chart
| Category | obes | smoking | hypertension | lga sp >2sd | sga sp <2sd |
|---|---|---|---|---|---|
| 0 | 20.0 | 20.0 | 6.0 | 5.7 | 14.3 |
| 1 | 34.1 | 29.3 | 1.0 | 7.4 | 2.5 |
| 2 | 32.9 | 32.9 | 1.0 | 7.1 | 0.0 |
| 3 | 44.9 | 33.1 | 2.0 | 8.7 | 2.4 |
| 4 | 58.0 | 20.0 | 8.0 | 10.0 | 4.0 |
| 5 | 68.8 | 25.0 | 6.0 | 13.3 | 0.0 |Maternal overweight/obesity
Prevalence, %
Maternal smoking
LGA
Gestational hypertension
SGA
2002-2004
Year
2011-2013
2014-2016
2005-2007
2008-2010
2017-2019
N=50
N=16
N=70
N=127
Data
N=35
N=82
